# Unraveling the antifungal and aflatoxin B1 inhibitory efficacy of nano-encapsulated caraway essential oil based on molecular docking of major components

**DOI:** 10.1038/s41598-025-95557-y

**Published:** 2025-04-29

**Authors:** Zeinab K. Hamza, Nivien A. Abosereh, Rasha G. Salim, Ahmed F. El-Sayed, Soher E. Aly

**Affiliations:** 1 Food Toxicology and Contaminants Department, Food Industries and Nutrition Institute, National Research Centre, Giza, Egypt; 2 Microbial Genetics Department, Biotechnology Research Institute, National Research Centre, Giza, Egypt; 3 Egypt Center for Research and Regenerative Medicine (ECRRM), Cairo, Egypt; 4 Molecular Modeling and Spectroscopy Laboratory, Centre of Excellence for Advanced Science , National Research Centre, Giza, Egypt

**Keywords:** *Aspergillus flavus*, Aflatoxin, Caraway essential oil, Gene expression, Molecular docking, Nanobiotechnology, Environmental biotechnology, Biotechnology, Environmental sciences

## Abstract

*Aspergillus flavus* and its secondary metabolites, particularly aflatoxin B (AFB_1_), pose significant threats to global crop production. Essential oils are considered excellent antifungal agents; however, their volatile nature and oxidative instability limit their practical use. This research aimed to develop a chitosan nanoemulsion loaded with caraway essential oil through the ionic-gelation process, utilizing sodium tripolyphosphate as a cross-linker. The nanoencapsulation was characterized using SEM, DLS, and FTIR analyses. The CEO-CSNPs inhibited *A. flavus* (35 mm at a concentration of 1000 ppm) and reduced aflatoxin production by 92.84% compared to free CEO. Down regulation of the relative expression of aflatoxin genes (*aflaD, aflaR*, and *aflaS*) in the aflatoxin biosynthesis pathway demonstrated its anti-aflatoxigenic mechanism. Molecular docking studies revealed that carvone and D-limonene exhibited strong binding energies for the three enzymes of *A. flavus*. These compounds showed decreased binding energies and various interactions at the active sites of important enzymes, suggesting their potential to inhibit these regulatory enzymes and consequently suppress AFB_1_ production.

## Introduction

Contamination of food and feed with mycotoxin is an unavoidable global challenge, posing posing severe food safety and economic issues worldwide. Aflatoxins (AFs) are among the most toxic and prevalent mycotoxins, produced primarily by *Aspergillus flavus* and *Aspergillus parasiticus*. AF contamination not only threatens human and animal health due to its carcinogenic, mutagenic, and immunosuppressive properties^1^ but also leads to significant economic losses by reducing crop yields, impacting trade, and increasing food insecurity worldwide^2^. AFs are significant contributors to the spoilage and biodeterioration of around one-fourth of global food crops, further exacerbating food security challenges^3^. It is frequently found in cereals, oilseeds, spices, meats, and many other foods and feeds (including dairy products), produced by fungal species of the genus Aspergillus^4^. Among the 18 known AF types, AFB1 is the most potent hepatocarcinogen and has been classified as as a group I carcinogen by the International Agency for Research on Cancer^5^.

Given the adverse effects and regulatory restrictions associated of synthetic preservatives, essential oils (EOs) and their bioactive components have duly received great attention as promising eco-friendly food preservatives and feasible alternatives against mycotoxin. EOs are classified as Generally Recognized as Safe (GRAS)^6^ by regulatory agencies^7,8^. *Carum carvi* L., known as caraway, is a biennial plant with fine and grooved stems from the Apiaceae family.

The pharmacological effects of caraway essential oils are well-established and proven. CEO exhibits antimicrobial, antioxidant, antiacetylcholinesterase, antidiabetic, analgesic, anti-inflammatory, antianxiety, antihyperglycemic, and antispasmodic properties, making it a potential candidate for combating fungal contamination and mycotoxin production in food systems^9^. The free form of EO remains a challenging task in practical food systems due to several critical factors such as inherent volatility, poor water solubility, and instability to environmental factors such as light, temperature, moisture, and possible interaction with food ingredients during storage^10,11^. Moreover, the need for high concentrations of essential oils (EOs) to achieve effective antifungal activity,which may negatively affect the organoleptic properties of the food, leading to consumer rejection of the product^12^.

In this context, nanoencapsulation using natural polymers has emerged as a promising strategy to address these limitations. Thus, the current investigation hypothesized that encapsulating caraway essential oil (CEO) in a natural, biodegradable polymeric nanocarrier, such as chitosan (CS), could overcome the challenges associated with free EOs and enhance their antifungal activities. In this study, chitosan was selected as the encapsulant due to its gelling ability, high entrapment efficiency, stability, and antifungal properties^13–15^.

This study hypothesizes that encapsulating CEO in a natural chitosan-based nanocarrier can enhance its antifungal efficacy and mitigate aflatoxin contamination in food systems. The study aims to (i) characterize the bioactive components of CEO using GC–MS, (ii) develop and assess the physicochemical properties of CEO-loaded chitosan nanoparticles (CEO-CSNPs), (iii) evaluate their antifungal and aflatoxin inhibition potential against *A. flavus*, and (iv) investigate their mechanism of action at the molecular level through gene expression analysis of aflatoxin biosynthetic pathway genes. Additionally, molecular docking analysis was performed to assess the interaction of major CEO compounds, (-)-Carvone and D-Limonene, have a robust affinity for the *aflS* protein of *A. flavus*.

## Experimental

### Chemicals and solvents

Sigma Aldrich Co. (USA) supplied sodium tripolyphosphate (TPP), Tween 80, 2,2-DiPhenyl-1-Picryl Hydrazyl (DPPH), microbial growth medium, and dichloromethane. Chitosan of medium molecular weight (CAS: 9012-76-4 with MW = 200–310 KDa and degree of deacetylation =  > 80%) was purchased from Sigma Aldrich Co. (St Louis, MO, USA).

### Fungal strains

*Asperagillus flavus* 3357 was supplied by the Northern Regional Research Laboratory (NRRL), Illinois, USA.

### Caraway essential oil (CEO) isolation

The essential oil was obtained from the disintegrated caraway fruit (100 g) by Clevenger- hydrodistillation for 4–6 h and stored in a dark bottle at 4 °C until analysis. The chemical composition of the extracted oil was evaluated using Varian gas chromatography/mass spectrometry (Perkin Elmer Auto XL GC Waltham, MA, USA). Individual compounds were identified according to their retention time and spectrum peaks that matched the published data.

### Encompassment of caraway oil (CEO) into chitosan (CS) nano-emulsion

Chitosan was dissolved in 0.1M glacial acetic acid and stirred overnight to get 0.2% of chitosan solution. Tween 80 (0.45 g) was added to 40 mL of chitosan solution and stirred continuously at 50 °C for 1.5 h then cooled to a room temperature. subsequently, 0.04 g of caraway essential oil (CEO) was added dropwise to 10 mL of the chitosan solution while stirring continuously for 30 min to obtain an oil-in-water emulsion with a chitosan-to-oil weight ratio of 1:2.

#### Ionic gelation process

3 mL of 0.02% (w/v) sodium tripolyphosphate (TPP) solution was gradually added to 3 mL of the oil-in-water (O/W) emulsion while stirring at 400 rpm for 40 min. The mixture was then centrifuged at 10,000 rpm for 30 min, and the resulting nanoparticles (NPs) were washed several times with deionized water. The NPs were subsequently freeze-dried and stored in the refrigerator until further analysis.

The encapsulation efficiency and loading capacity of the developed formula were determined using a UV–vis spectrophotometer, following the method outlined by Hosseini et al.^16^. The concentration of CEO in the chitosan-based nanoparticle was measured at 244 nm, the wavelength of maximum absorption for CEO, using a calibration curve of free CEO in ethanol. The formula without CEO served as the blank. The EE % and LC % were calculated using the following equation.

$${\text{EE }}\% = \, \left( {{\text{Trapped \, CEO \, mass}} - {\text{ free\, unloaded CEO}}} \right)/ \, \left( {\text{Initial \, CEO\, mass}} \right) \, \times { 1}00$$

$${\text{LC }}\% \, = \, \left( {\text{Trapped\, CEO\, mass}} \right)/ \, \left( {\text{freezev dried\, sample\, weight}} \right) \, \times { 1}00$$

### Physicochemical characterization

The average diameter, size distribution, and zeta potential of CEO-CSNPs were evaluated using dynamic light scattering (DLS) with a Zetasizer™ 3000E (Malvern Instruments, Worcestershire, UK). The polydispersity index (PDI) of the CEO-CSNPs was determined via Malvern software employing an exponential sampling method. Prior to zeta potential measurements, the samples were subjected to sonication. Each measurement was repeated three times to ensure accuracy.

The morphological features of the synthesized CEO-CSNPs was determined using scanning electron microscope (Quanta FEG 250 SEM, Tokyo, Japan).

FTIR spectra of chitosan, chitosan NPs, free CEO, and encapsulated CEO were recorded using an FTIR spectrometer (Thermo Fisher Scientific Inc., USA). The spectra were acquired with 16 scans at a resolution of 4 cm^−1^, covering a wavenumber range from 500 to 5000 cm^−1^.

### Antifungal activity assay of caraway essential oil (CEO) and caraway-chitosan nanoparticles (CEO-CSNPs)

The antifungal activity of CEO and CEO-CSNPs was assessed using the agar well diffusion assay^17^. A standardized inoculum of the test fungi (10 μL) was spread onto Potato Dextrose Agar plates. Wells, 6 mm in diameter, were punched into each plate, and 100 μL of various concentrations of CEO (1000, 500, 250 ppm) or the solvent (DMSO 1%) as a control were added to each well. The plates were incubated at 25 °C for 3 days. The zone of inhibition was measured and recorded as the average (n = 3) and expressed in millimeters. All data were presented as mean ± SD, with experiments performed in triplicate.

### Efficacy of caraway essential oil (CEO) and caraway-chitosan nanoparticles (CEO-CSNPs) as inhibitor of aflatoxins secretion

The ability of CEO and CEO-CSNPs to inhibit aflatoxin production was assessed according to Kumar et al.^18^. Various concentrations (1000, 500, 250 ppm) were prepared and each flask was inoculated with 25 μL of a spore suspension (10^6^ spores/mL) of a toxigenic strain of *A. flavus* and incubated at 25 ± 2 °C in a BOD incubator for 15 days. Potato dextrose broth medium with 25 μL of *A. flavus* suspension without treatment was used as a blank. After the incubation period, the mixtures were filtered, and the filtrates were extracted with 50 mL of chloroform using a separating funnel, followed by dehydration with anhydrous sodium sulfate. The extracts were then evaporated at 50 °C under vacuum. The dried residues were dissolved in 2 mL of methanol for aflatoxin quantification using Agilent C18 (4.6 × 250 mm i.d., 3.5 μm) HPLC. The mobile phase consisted of water, methanol, and acetonitrile in a 6:3:1 ratio, with a flow rate of 1 mL/min. The injection volume was 20 μL for AFG_1_ and AFB_1_ (at 50 ng/mL) and 15 μL for AFG_2_ and AFB_2_ (at 15 ng/mL). The fluorescence detector was set to an excitation wavelength of 360 nm and an emission wavelength of 450 nm, with the column temperature maintained at 40 °C.

### Isolation of total RNA

Total RNA was isolated with the RNeasy Mini Kit (Qiagen, catalog number 74104c). Ten, the Rever Aid First Strand cDNA synthesis kit was used for cDNA synthesis (Termo Scientific cat. no. K1621). According to the manufacturer’s instructions, all experiments were carried out. The reaction tubes were rapidly chilled in an ice room before being utilized for DNA amplification using qRT-PCR.

### Quantitative real time-polymerase chain reaction (qRT-PCR)

Applied Biosystems’ StepOne™ real-time PCR equipment (Thermo Fisher Scientific, Waltham, MA USA) was used to analyze fungal gene transcripts in both control and treatment samples. Gene-specific primers were designed using sequencing results from various genes, and qPCR was performed using the Primer3 software (http://frodo.wi.mit.edu/cgi-bin/primer3/primer3_www.cgi) as shown in Table 1. PCR reactions were done in 25 µL reaction mixtures comprising 12.5 µL of 1 × SYBR® Premix ExTaqTM (TaKaRa, Biotech. Co. Ltd.), 0.5 µL of 0.2 µM sense primer, 0.5 µL of 0.2 µM antisense primer, 6.5 µL distilled H2O, and 5 µL of cDNA template. The reaction program was spectated into three phases. First, it started at 95 °C for 3 min. The second is 40 cycles in which each cycle is divided into three steps: (a) 95 °C for 15 s (b) 55 °C for 30 s; and (c) 72 °C for 30 s. Third, 71 cycles were initiated at 60 °C, with the temperature increasing by around 0.5 °C every 10 s until it reached 95 °C. Each experiment has a distilled water control. GAPDH (housekeeping gene) served as a control gene for differences in total cDNA input between samples. At the completion of each qRT-PCR, a melting curve analysis at 95° was done to check the primers’ quality. Each experiment had three replicates. The relative amount of the genes of interest was assessed according to Ruijter et al.^19^.

**Table 1:** The primers used in qRT-PCR of *aflD*, *aflS*R, and *aflS.*

Primers name	Primers sequence (5′ → 3′)
*AflD* F	CTGACGGCGTACGGAGTGTC
*AflD* R	GAGCACAGATGCCTGCCACA
*aflR* F	GGATGAGGAAGACCAGCCGC
*aflR* R	GAGCGAGGGCAACAACCAGT
*aflS* F	GGCCGAAGATTCCGCTTGGA
*aflS* R	GAGCGAGGGCAACAACCAGT

### Molecular modeling and computational analysis of aflDand *aflS* proteins:

#### Secondary structure prediction of *aflS *and *aflD* proteins

The NCBI protein sequencing database was the source of the primary sequences for the *aflS* and *aflD* proteins of the *A. flavus* strain. To align these protein targets, the Clausal Omega program was utilized with its default parameters^20^.

#### Molecular modeling and model validation

The comparative modeling method was employed to construct a 3D representation of the *aflR*, *aflS*, and *aflD* proteins by templating amino acid sequences from closely related homologues with known X-ray crystal structures. Modeller 9v20 facilitated the generation of the 3-D structure. Utilizing the established crystal structure as a guide, homology models were developed. A total of three hundred 3D models were generated and assessed using Modeller software following manual adjustments to the template and query sequence alignment files. The protein preparation wizard was utilized to implement a force field for energy minimization within the created models. The accuracy of the initial model was scrutinized to ascertain the credibility of the modeled structure^20^.

#### Molecular docking of *aflR*, *aflS *and *aflD*with ligands

The 3D models of the *aflR*, *aflS* and *aflD* utilized in the docking investigation were created using Modeller and their respective PDB identification codes. Hydrogen atoms were added to the receptor molecule using Autodock Vina software’s MG Tools^21^. The preceding steps were performed for each protein and saved for molecular docking. An Open Babel was used to convert the compounds into a mol2 format^22^. Autodock tools were used to convert the ligand molecules into dockable *pdbqt* format. A ligand center maps with a spacing of 0.375 A° and grid dimensions of 90 A° × 90 A° × 90 A° was produced by the AutoGrid program. The binding interactions between these ligands with 3-D model of aflR, *aflS* and *aflD* were visualized by PyMol package. All the docking experiments were done using Autodock Vina 1.0^21^. Also, The Discovery Studio 4.5 program was used to explain 2-D hydrogen bond interaction.

### Statistical analysis

All the values are presented as means ± standard deviation of the means (SD). Comparisons between different groups were carried out using one-way analysis of variance (ANOVA) followed by Tukey’s multiple comparison test for multiple comparisons. Graphpad Prism software version 5 (GraphPad Software, La Jolla, CA) was used to carry out these statistical tests. The difference was considered significant when *p* ˂ 0.05.

## Results & discussion

### Caraway essential oils isolation

**Fig. 1:**
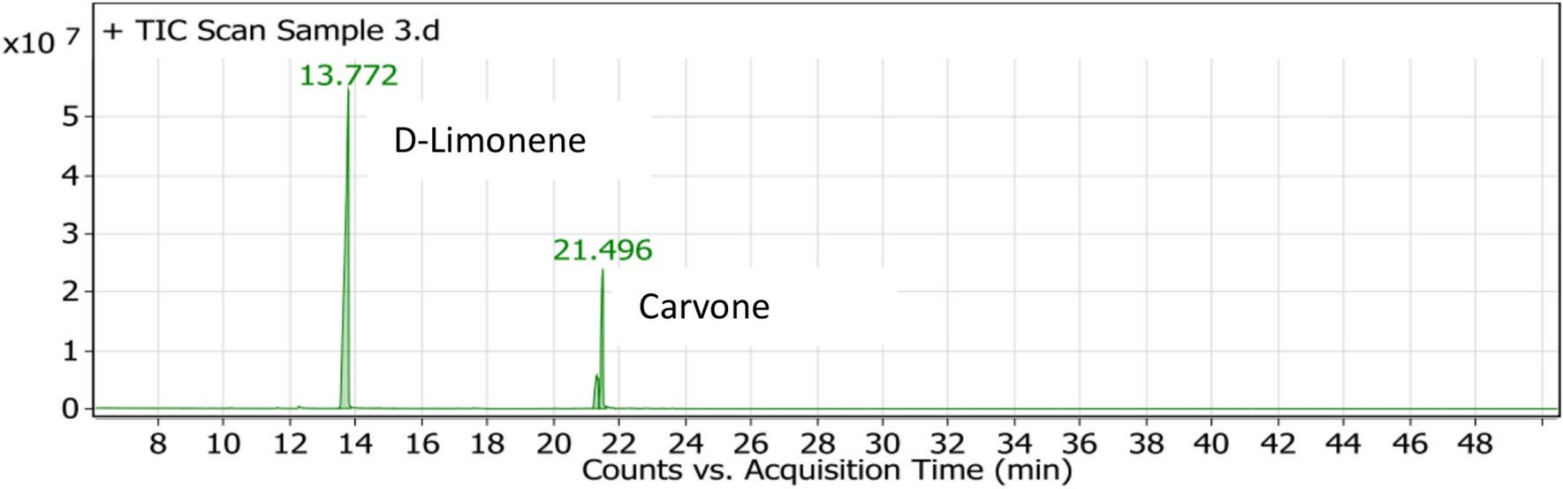
GC–MS chromatogram of the bioactive compounds present in caraway essential oil.

CEO had been isolated and characterized chemically through GC–MS which showed D-Limonene (73.86%) and (-)-Carvone (26.14%) as major components of CEO (Fig. 1, Table 2). The variations in the percent composition in CEO are attributed to the differences in extraction procedures, plant parts utilized, harvesting time, plant age, thereby, affecting the chemical composition of CEO^23^. Hence, prior to the investigation of antifungal and anti-aflatoxigenic efficacy of the studied oil, the chemical standardization of EOs is an important parameter of the present research.

**Table 2:** The chemical composition of CEO (major active compounds of CEO).

Peak	RT	Name	Formula	Structure	Area sum %
1	13.772	D-Limonene	C_10_H_16_		73.86
2	21.319	(-)-Carvone	C_10_H_14_O		26.14%

To estimate the amount of CEO encapsulated in the CSNPs, UV–Vis spectroscopy was employed. The concentration of encapsulated CEO was determined by measuring its absorbance at 244 nm. A calibration curve was constructed for pure caraway essential oil (ranging from 100 to 700 µg/mL) in ethanol, which was used to calculate the encapsulation efficiency (EE) and loading capacity (LC) of the CEO-CSNPs. The calibration curve for CEO showed an R^2^ value of 0.986. The percentage amount of CEO encapsulated in CSNPs was estimated spectrophotometrically based on the calibration curves at 244 nm (the maximum absorption of CEO). The EE % of CEO in CSNPs was determined 84.959% while LC % was 2.484. The concentration of the evaluated nano-encapsulated EO from the initial amount represents (EE) whereas, (LC) represents the EO concentration in a constant amount of chitosan NPs. These findings are are consistent with the results of Upadhyay et al.^24^ and Shetta et al.^25^, which found that the highest loading capacities were obtained at higher concentrations of essential oil and polymer during the preparation of chitosan nanoparticles containing *Cananga odorata* EO and peppermint/green tea essential oil, respectively. The efficient entrapment of CEO into chitosan biopolymer up to 85% promptly supports the superiority of chitosan matrix to outstandingly protect the loaded CEO from the degradation under the fluctuating environmental conditions. Consequently, the efficient entrapment and imprisoning of volatile constituents of CEO will amplify the fungal inhibition and extend the maximum product shelf-life.

**Fig. 2:**
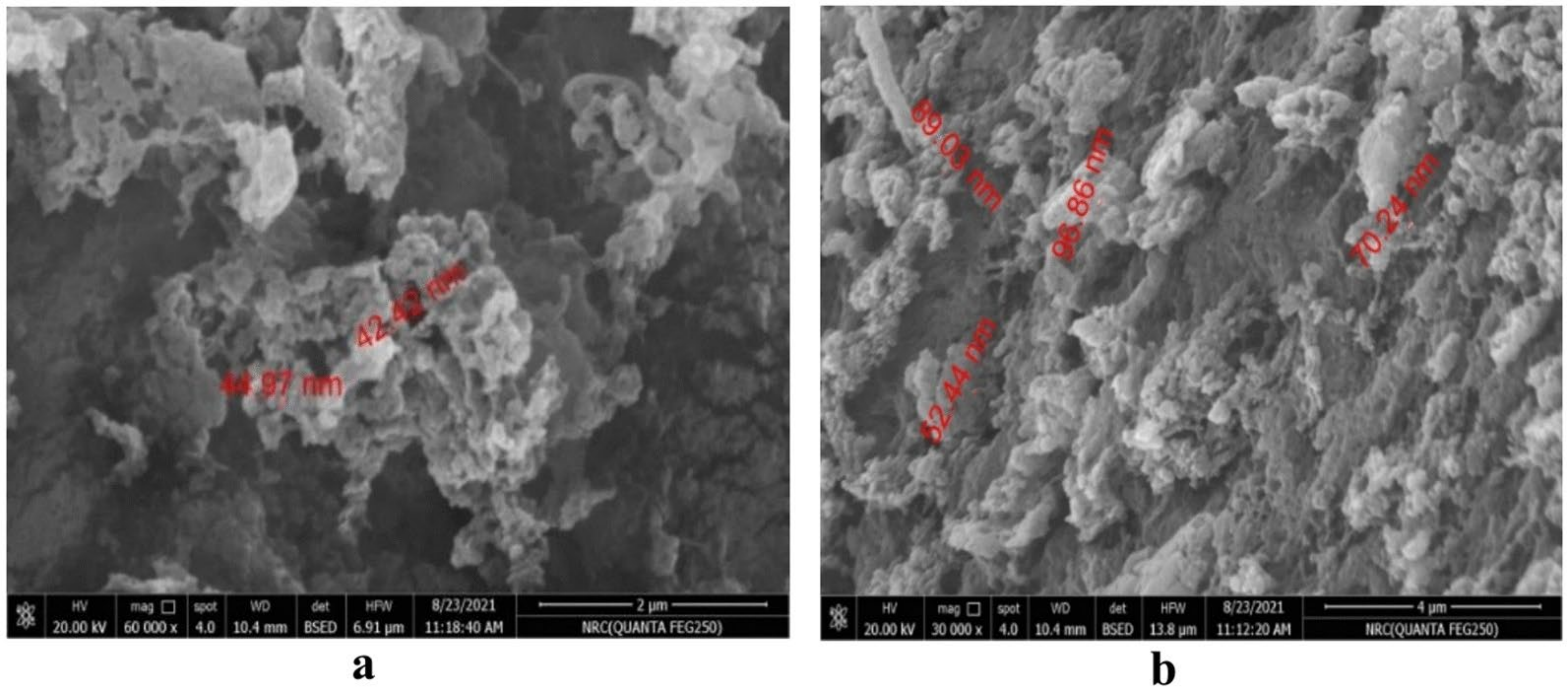
SEM of the unloaded (**a**) and loaded CEO-CSNPs (**b**) at about 8000 × and 30,000 × magnifications.

**Fig. 3:**
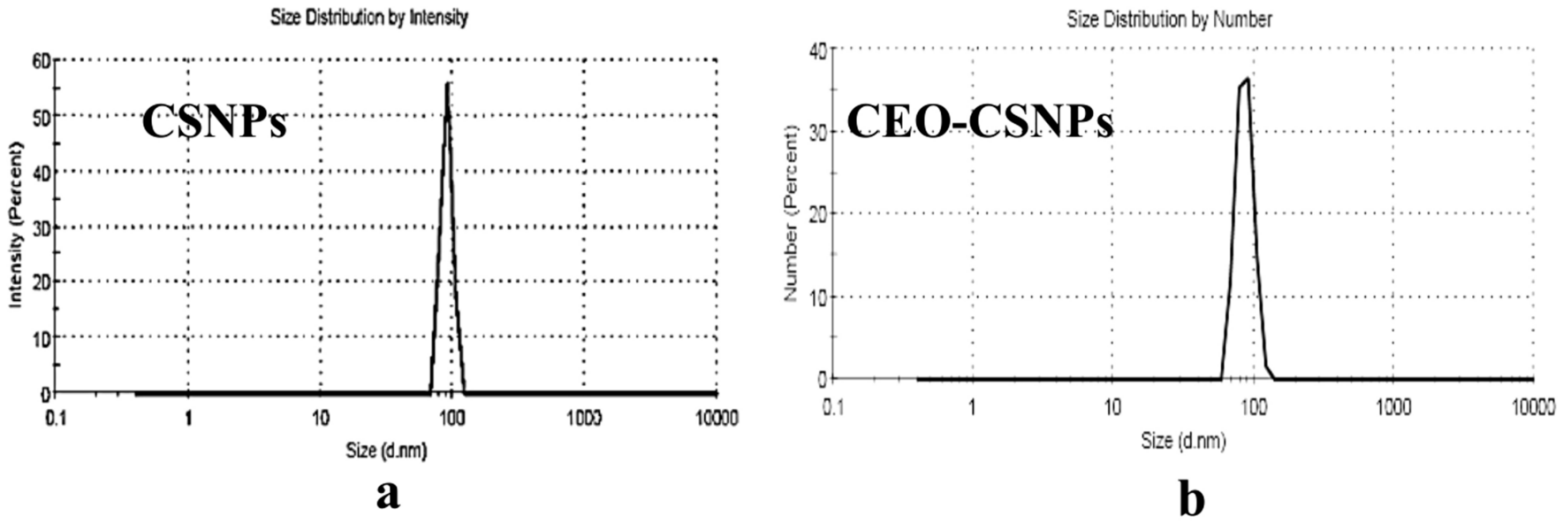
The size distribution profile of CSNPs (**a**) and CEO-CSNPs formulation (**b**) by number.

**Fig. 4:**
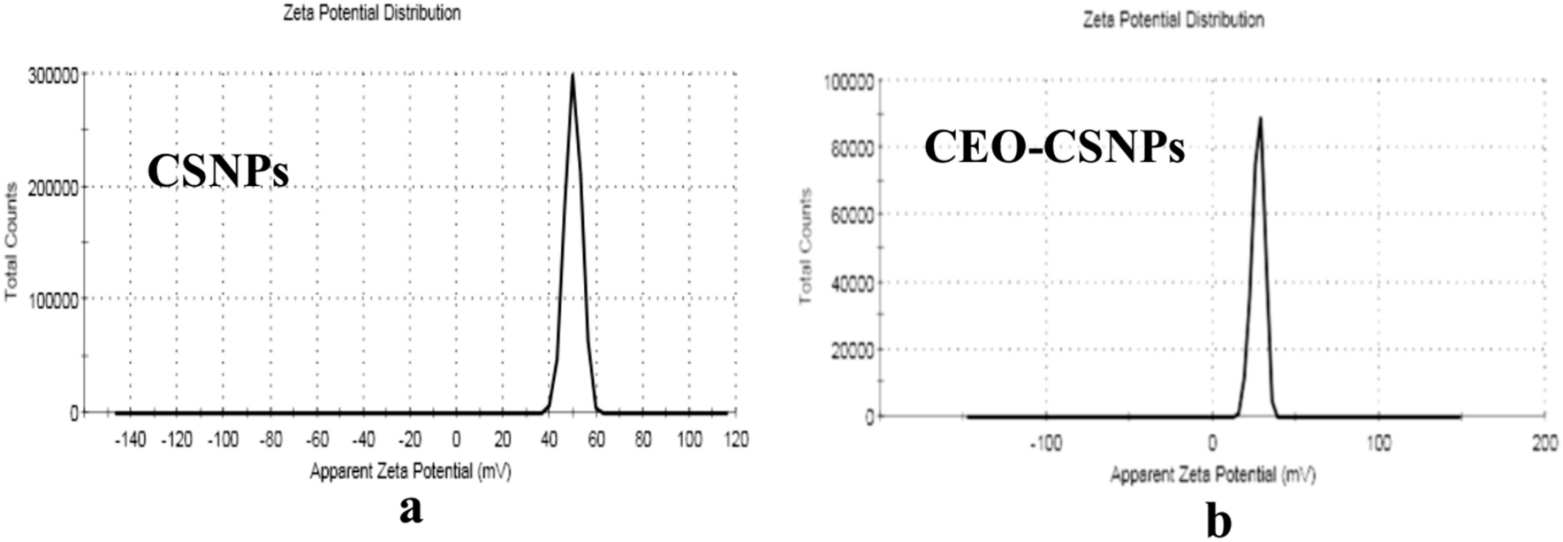
The Zeta potential value of CSNPs (**a**); and fabricated CEO-CSNPs (**b**).

In the current study, The zeta size (ZS), zeta potential (ZP), and morphology of CEO-CSNPs were analyzed using SEM techniques. The chitosan nanoparticles (CS-NPs) exhibited a spherical shape (Fig. 2) with an average size of 11.24 ± 1.73 nm (Fig. 3a) and a zeta potential of 42.42 mV (Fig. 4a). In comparison, the CEO-CSNPs also displayed a spherical shape (Fig. 2b) but were larger, with an average size of 86.77 ± 12.07 nm (Fig. 3b) and a zeta potential of 27.7 ± 3.79 mV (Fig. 4b). The polydispersity index (PDI), which ranged from 0.103 to 0.421, indicated that the CEO-CSNPs were mono-dispersed with a uniform particle distribution (Table 3).

**Table 3:** DLS based analysis of average particle size, zeta potential and the poly-dispersity index for CSNPs and CEO-CSNPs.

Sample	Particle size (nm)	Zeta potential (Mv)	PDI
CS NPS	11.24 ± 1.73	49.4 ± 3.56	0.463
CEO CSNPs	12.07 ± 86.77	27.7 ± 3.79	0.668

The size distribution observed from SEM and DLS suggested that the size of CEO-CSNPs was larger than the CSNPs, possibly due to the entrapment of the CEO into CSNPs (Table 3). The larger particle size observed from DLS measurements could be attributed to nanoparticle swelling and aggregation, in contrast to the smaller sizes obtained from SEM, where the samples were dry-prepared and measured individually^26^. According to Noghabi^27^, low polydispersity displays uniformity of diameter. The given results of CEO-loaded CSNPs show the particles are distributed in the suspended medium with uniform size.

As shown in Fig. 4a, the ZP of CS NPs was + 49.4 mV. In contrast, the ZP of CEO-CSNPs decreased to + 27.1 mV (Fig. 4b, Table 3). The results demonstrated that loading CEO significantly reduced the ZP of CS from + 49.4 mV to + 27.1 mV (Table 3). According to previous research, zeta potentials above 60 mV indicate good stability, while values between 30 and 20 mV suggest physical and limited stability, respectively and Zeta potentials below 5 mV are associated with agglomeration^28^. These findings are consistent with those reported by Upadhyay et al.^24^. In our study, the reduction in ZP observed in CEO-CSNPs could be attributed to a shielding effect, where the CEO coats the protonated NH2 groups on the CSNPs. The decrease in the ZP value to + 27.1 mV in CEO-CSNPs may increase the attraction between the nanoparticles, potentially explaining the observed increase in their average diameter.

### FT-IR analysis

**Fig. 5:**
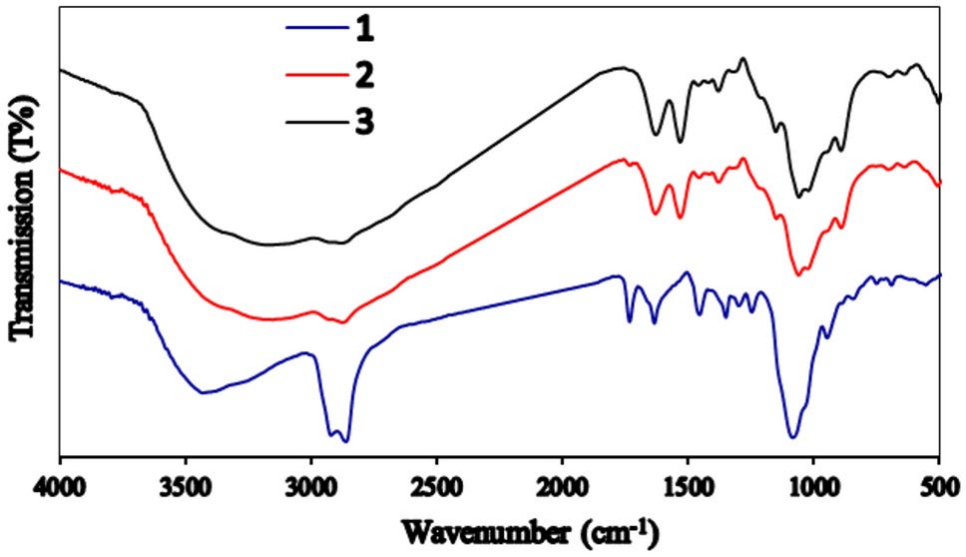
Fourier transforms infrared spectroscopy (FTIR) spectra of the following: 1. Chitosan, 2. Chitosan NPs, 3. CEO-CSNPs.

Figure 5 shows the FTIR analysis of powdered chitosan (1), CSNPs (2), and CEO-CSNPs (3) after encapsulation. Typically, chitosan powder exhibits specific peaks at 3482 cm^–1^, indicating the presence of hydroxyl groups and –NH_2_ stretching, and a peak at 1665 cm^–1^ associated with amide I. The FTIR spectrum also shows a glucose ring at 889 cm^–1^, characteristic of chitosan. In the spectrum of CEO-loaded chitosan-TPP nanoparticles, the characteristic absorption bands appear at the same wavenumbers as the stretching vibrations of (PO_3_) groups and (P=O). Compared to the CS-TPP NPs spectrum, the presence of CEO increased the intensity of the C–H stretching peak at 2866–2925 cm^–1^, confirming that CEO is encapsulated in the chitosan nanoparticles. Additionally, the N–H_2_ bending peak of amide II shifted from 1585 to 1571 cm^–1^, and new peaks emerged at 1088 and 1251 cm^–1^.

### In vitro antifungal activity of CEO and encapsulated CEO

**Fig. 6:**
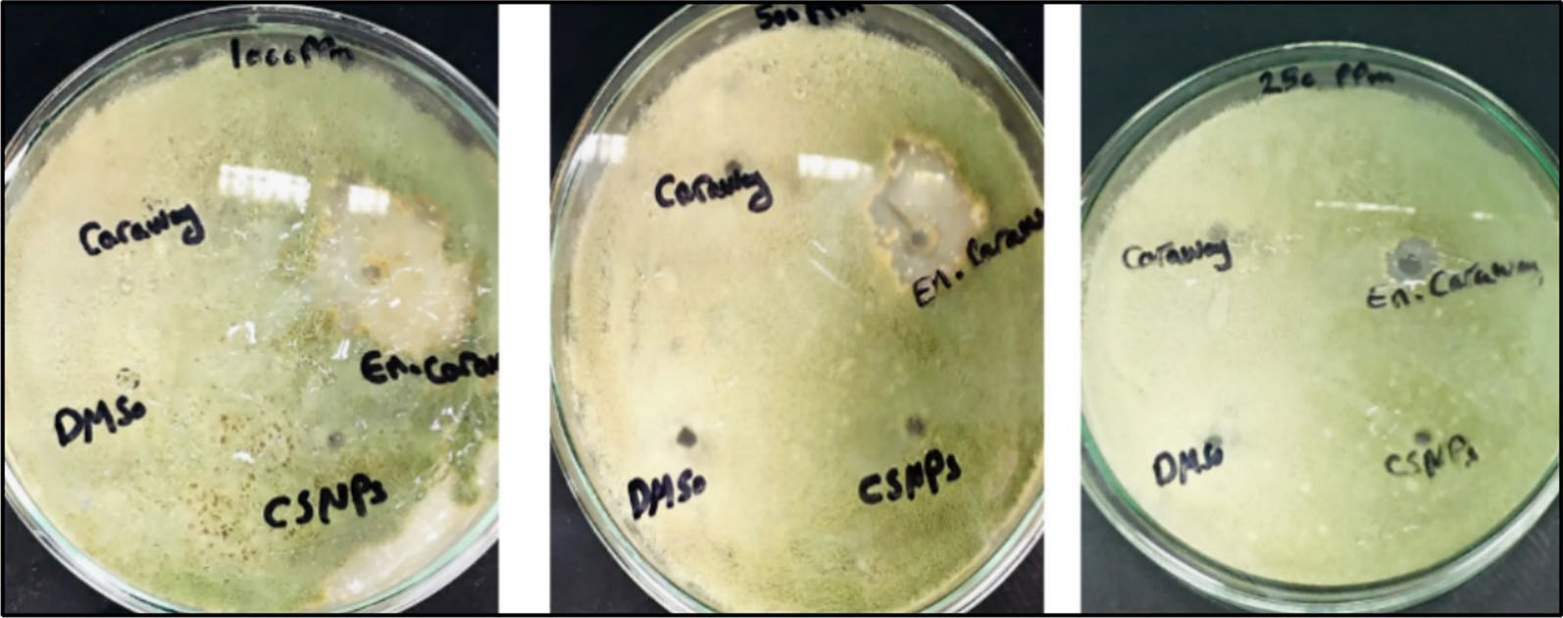
In vitro antifungal activity CEO and encapsulated CEO under in vitro conditions against *A. flavus.*

The in vitro antifungal activity of CEO and CEO-CSNPs was assessed using the zone of inhibition on *A. flavus* mycelial growth after 7 days of incubation. The difference in the antifungal activity of CEO before and after encapsulation was investigated by the agar disk diffusion method, the negative control was DMSO (1%). The CEO encapsulated in chitosan nanoparticles showed the highest antifungal activity with an average inhibition zone of 35 mm at 1000 ppm compared to the free CEO (Fig. 6). Interestingly, sporulation plays a crucial for aflatoxin production. Therefore, inhibiting sporulation at 1000 ppm may reduce AFB1 secretion by *A. flavus*.

The findings show that CEO-CSNPs have antifungal activity against *A. flavus*, which can be attributed to to Van der Waals forces and hydrogen bonding between the negatively charged phospholipids of the fungal cell membrane and the positively charged CEO-CSNPs.This interaction can destabilize the membrane, affecting the characteristics and eventually killing the microorganisms^29^. These results agree with Das et al.^30^ who found an improvement of the carvone-nanoemulsion in inhibition of *A. flavus* (0.5 µL/mL) and AFB_1_ production (0.4 µL/mL) more effectively than unencapsulated carvone. This suggests the possibility of synergistic antifungal effect among the individual components. Furthermore, the fabrication of CSNPs by ionotropic gelation makes advantage of chitosan’s cationic charged amino groups for covalent link formation with S-TPP, which most likely reduces antifungal effectiveness by modifying the fungal cell’s membrane fluidity.

### Efficacy of EOS as inhibitor of aflatoxins (AFB_1_, AFB_2_, AFG_1_ and AFG_2_) secretion by *A. flavus*

The effects of different unloaded and loaded CEO essential oil concentrations (250–1000 ppm) upon AFs production by *A. flavus* were studied to assess the fungitoxicity and AFs inhibitory efficacy of unencapsulated and nano-encapsulated CEO*.* Chitosan nanoemulsion exhibited negligible fungitoxic while loaded CEO inhibited the growth of assayed fungi than those observed for pure CEO. The decrease in fungal growth and AFs production was completely dependent on the concentration of nanoencapsulated CEO, which showed a dose-dependent inhibitory pattern. Several research have been published on the antimicrobial activity of chitosan. However, our results contradict earlier findings and may point to a unique synergistic action of chitosan matrix with CEO in inhibiting fungal cell proliferation and AFB_1_ release.

The enhanced antifungal and anti-aflatoxigenic activity of CEO loaded chitosan nano-emulsion compared to unencapsulated CEO is likely due to the nano-sized particles and the controlled release of volatile components. The inhibition of fungal growth may result from effects on various stages of aflatoxin biosynthesis and secretion. This could include the suppression of critical metabolic intermediates like norsolorinic acid, disruptions in toxin transport due to an unfavorable proton motive force in the membrane, or alterations in carbohydrate metabolism that impact aflatoxin production^31^. Furthermore, Guzmán-de-Peña and Ruiz-Herrera^32^ elucidated a strong correlation between aflatoxin biosynthesis and sporulation in Aspergillus parasiticus, demonstrating that inhibiting sporulation leads to a suppression of aflatoxin production^32^. In the same way, other research has found that less sporulation is linked to less aflatoxin production, even when mycelial growth is not affected^33^. These results suggest that the control pathways for sporulation affect aflatoxin production. This shows how complex the relationship is between the growth of fungi and secondary metabolism.

Gniewosz et al.^34^ used caraway oil against *A. niger* at a concentration of 0.12% to minimize the bioburden of *A. niger* on immature roots of the carrot plant. A lot of research has attribute the CEO’s bactericidal effect against a wide range of pathogenic fungus and bacteria to its two major components, carvone and limonene^35^. Both carvone and limonene have a strong affinity for membranes, disrupting the membrane barrier function. Other investigations have shown that limonene inhibits the growth of a wide range of bacteria^35^.

In the present study caraway essential oil contain more limonene compare to carvone (1.5 fold) in GC analysis; and accordingly to the previous studies and from an economic standpoint more extensive possibilities of the application in the biotechnological and medical fields can be aroused by CEO-CSNPS in future as a green bio nanocomposite alternative instead of synesthetic agrochemicals^36^.

### Quantification of aflatoxins by high-performance liquid chromatography

**Fig. 7:**
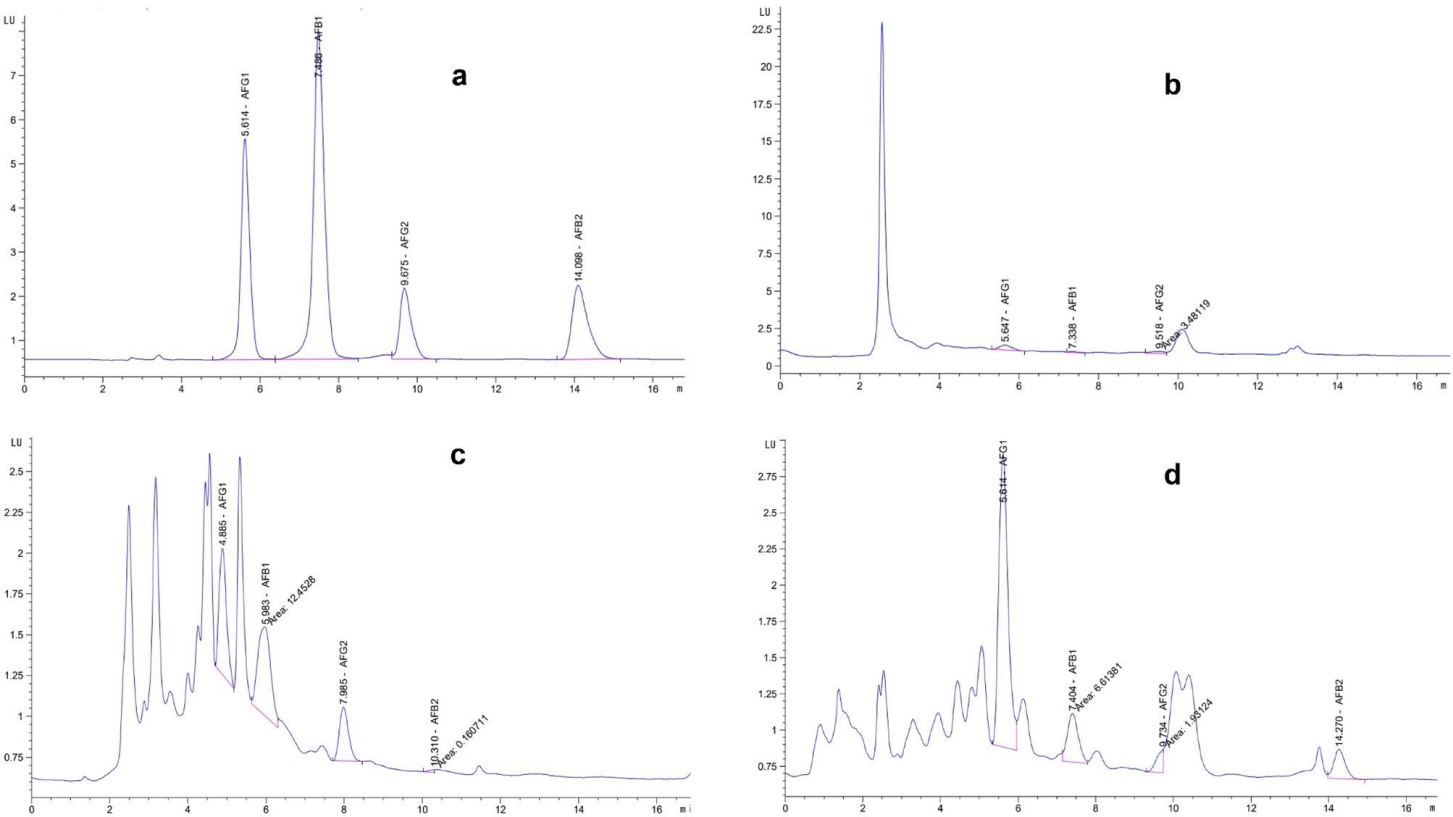
HPLC chromatogram of the standard AF mixture, including AFB_1_, AFG_1_ at 50 ng/mL, and AFB_2_, AFG_2_ at 15 ng/mL (**a**). The effect of encapsulated CEO treatment on the production of AFB_1_, AFB_2_, AFG_1_, and AFG_2_ by *Aspergillus flavus* is shown at CEO concentrations of (**b**) 1000 ppm, (**c**) 500 ppm, and (**d**) 250 ppm.

The essential oil concentration required to prevent aflatoxin (B_1_, B_2_, G_1_, G_2_, and total AFs) formation by *A. flavus* was found to be in the sub-MIC range (250 ppm, 500 ppm, and 1000 ppm). Figure 7 and Table 4 indicate the effects of CEO-CSNPS on mycelial mass and aflatoxin production. The results reveal that caraway essential oil has considerable inhibitory effects on *A. flavus*. Our findings indicate a synergistic interaction between CEOs and CSNPs that results in an enhanced effect in inhibition AFs synthesis (1.5, 1.2, 1.6 and 1.4-fold) on AFG_1_, AFG_2_, AFB_2_ and the total AFs at 1000 ppm relative to unloaded CEO, that show a moderate inhibitory effect on AFG_1_ and AFG_2_ synthesis (60.6 and 75%, respectively). It is believed that the active components of EOs hinder one of the steps in mycotoxin production. According to Omidbeygi et al.^37^, the constituents of EOs and extracts penetrate the cell membrane, integrating with membrane enzymes and proteins, causing the loss of macromolecules from the interior of the cell, causing to alteration and ultimately, death.

**Table 4:** Inhibitory effects of CEO on upon AFB_1_ and AFB*2*, AFG_1_ and AFG_2_ production by *A. flavus* before and after encapsulation**.**

CEO conc. (ppm)	AFs types				
	AFG_1_	AFB_1_	AFG_2_	AFB_2_	AFs
AFs inhibition % before encapsulation (unloaded CEO)					
1000 ppm	60.66923	72.66436	75.88805	60.30534	62.82739
500 ppm	59.04187	21.28028	37.45963	0	55.6
250 ppm	56.4637	0	25.94187	0	45.8
AFs inhibition % after encapsulation (loaded CEO-CSNPs)					
1000 ppm	91.93637	84.77509	97.20129	100	92.84
500 ppm	88.37082	55.19031	55.11302	96.18321	83.8
250 ppm	49.58859	28.2 0069	81.37783	93.12977	54.99

These results align with the studies by da Cruz Cabral et al.^38^, Prakash et al.^39^ and Powers et al.^40^ who demonstrated that the growth of mycotoxigenic fungi and their connected mycotoxins was effectively modulated by incorporating essential oils. These results support future studies into potential application of CEO-CSNPS to diverse types of food, as well as the selection of an efficient technique, such as directly incorporating them into the product, coating the product’s surface, dispersing them throughout, or integrating them into the packaging environment.

### Analysis the effect of caraway on *aflR, aflS *and *aflD* expression in aflatoxigenic *A. flavus* using qRT-PCR.

**Fig. 8:**
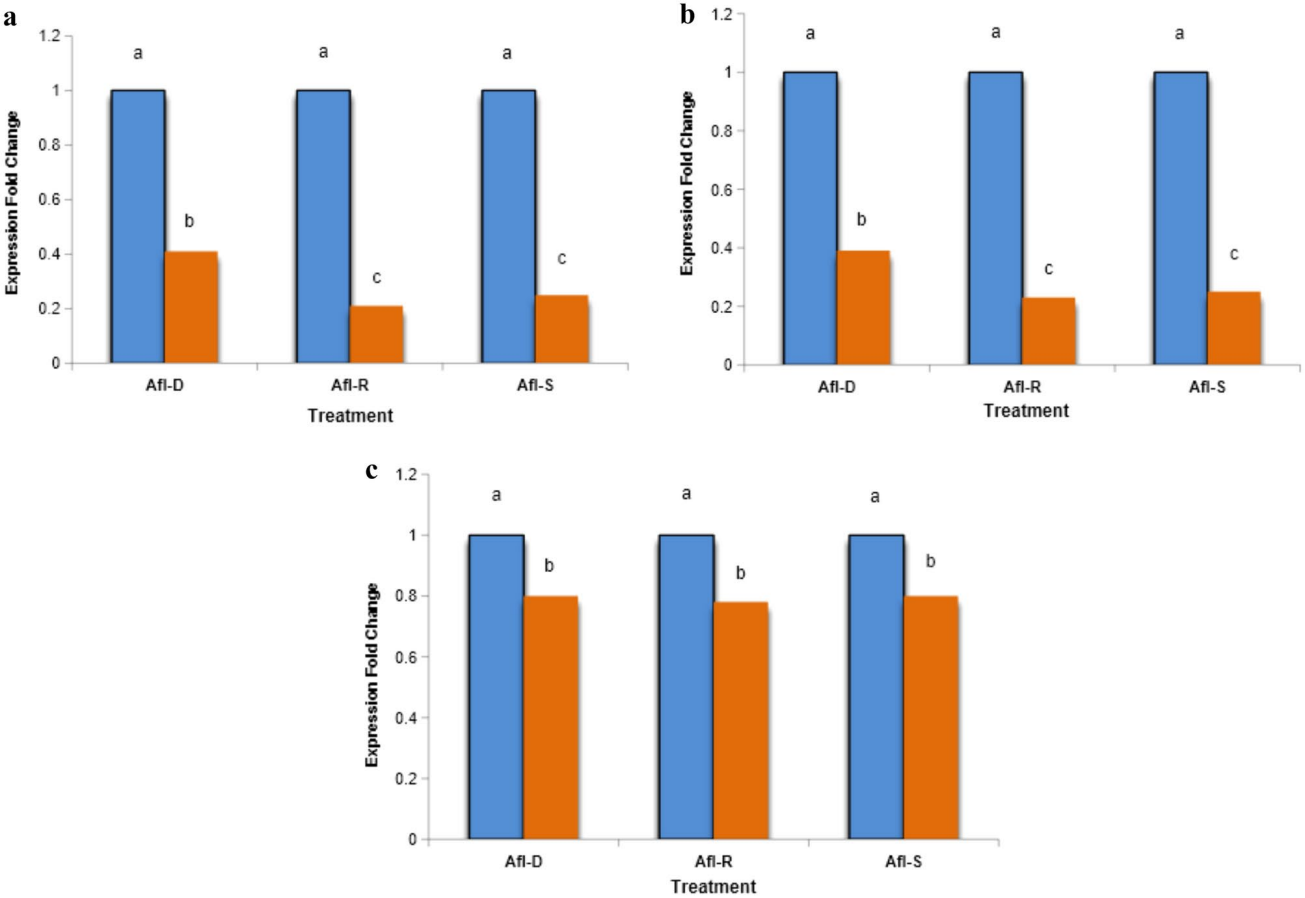
(**a**) The effect of caraway at 250 ppm on *aflR*, *aflS* and *aflD* expression in aflatoxigenic *A. flavus* using qRT-PCR. Different letters (a, b, c) indicate statistically significant differences between groups p ˂ 0.05. (**b**) The impact of encapsulated caraway at 250 ppm on *aflR*, *aflS* and *aflD* expression in aflatoxigenic *A. flavus* using qRT-PCR. Different letters (a, b, c) indicate statistically significant differences between groups p ˂ 0.05. (**c**) The impact of of capsule only chitosan NPs on *aflR*, *aflS* and *aflD* expression in aflatoxigenic *A. flavus* with qRT-PCR. Different letters (a, b) indicate statistically significant differences between groups p ˂ 0.05.

The purpose of this study was to assess the effects of CEO, encapsulated caraway, and capsule only chitosan NPs on the expression of two regulatory genes (aflR and *aflS*) and one structural gene (aflD) that were previously detected and sequenced in *A. flavus* (LC368455) which is listed in the Genbank database under accession numbers (LC537158, MW055253, and LC537157) respectively and are involved in the aflatoxin biosynthesis pathway. From Fig. 8a, the expression analysis of the transcripts of genes (*aflR, aflS* and *aflD*) in CEO treated aflatoxigenic *A. flavus* at 250 ppm indicated that the expression of these transcripts correlates with CEO treatment. Interestingly, the expressions of the three genes (aflR, *aflS*, and aflD) were significantly down regulated due to caraway treatment at a concentration of 250 ppm. Notably, the *aflR* gene expression showed marked downregulation due to both CEO treatment at 250 ppm and encapsulated caraway at the same concentration. Additionally, as shown in Fig. 8b, the expression of the regulatory genes *aflR*, and *aflS*, a long with the structural gene (*aflD*) involved in aflatoxin biosynthesis exhibited no changes at the treatment concentration of capsule only chitosan NPs as illustrated in Fig. 8c. The results demonstrated that the examined essential oils significantly inhibited fungal growth and aflatoxin production in *A. flavus*, with the extent of inhibition depending on the type and concentration of the essential oils used. Aflatoxin production is supported by an enzyme cascade consists of twenty-one steps, regulated in *A. flavus* by a gene cluster that includes the regulators aflR and *aflS*.^41^. The reduction of AFB1 production was linked to the decreased transcription levels of the aflatoxin cluster genes. Despite the interest of many researchers in investigating the anti-aflatoxigenic mechanism of caraway essential oil, the detailed molecular mechanisms behind aflatoxin biosynthesis inhibition by CEO remain largely unclear, possibly due to its constituents (Carvone and D-Limonene). Additional research has revealed that some natural compounds may exert an anti-aflatoxigenic effect by inhibiting the aflatoxin biosynthesis pathway through the reduction of the expression of aflatoxin biosynthetic genes (AFs). Indeed, natural phenols and terpenoids have been shown to decrease the expression of internal transcriptional regulators (*aflR and aflS*) in the aflatoxin biosynthesis pathway^42^. Additionally, plant-derived natural components could reduce the expression of structural genes such as *aflD, aflK, aflE, aflM, aflO, aflP, and aflQ*^43,44^ (Lv et al. 2018). However, the processes of their action at the molecular level still need to be fully elucidated.

### Molecular modeling and computational analysis of *aflD *and *aflS* proteins

#### Secondary structure prediction, homology modeling and validation of *aflD* and *aflS*

The inferred amino acid sequences from the *aflD* and *aflS* genes (protein ID BCD52744 and ULA70966.1) were aligned against the Protein Data Bank (PDB) using BLASTp to conduct a sequence similarity search and enable comparative modeling. The alignment of the aflDand *aflS* genes with their respective templates was carried out using the ESpript server, and the proteins’ secondary structures were predicted. The DSSP program characterized the aflDmodel, revealing a composition of 9 α-helixes and 7 β-sheets. In contrast, the *aflS* model exhibited 17 α-helixes, and 10 β-sheets.

Homology modelling process of the 3D structures of aflDand *aflS* from *A. flavus* was executed using Modeller 9v20. Following the generation of the model, energy minimization was performed utilizing the force fields from the YASARA Server in the Swiss PDB viewer. Subsequently, the created models of aflDand *aflS* underwent evaluation through PROCHECK, Verify3D, and ERRAT. The PROCHECK analysis of the *aflS* and aflDprotein 3D models revealed that 93.5% and 92.8% of residues occupied the most favored regions, with 6.5% and 7.2% in additionally allowed regions, respectively. Notably, no residues were found in generously allowed or disallowed regions of the Ramachandran plot, indicating the high quality of the generated models. Furthermore, the overall quality factor and the compatibility of the atomic models with the amino acid sequences (3D–1D) for the *aflS* and aflDproteins were determined as 87.42% and 93.68% for Verify3D, and 96.40% and 95.51% for ERRAT, respectively. These assessments, along with the Ramachandran plot, ERRAT, Verify-3D, and PROCHECK analyses, collectively affirm the credibility and high quality of the constructed models as illustrated in Tables 5 and 6.

**Table 5:** Plot statistics of template and generated models of *aflD* and *aflS* proteins:

Area	Afla-S protein				Afla-D protein			
	Statistics of template		Statistics of model		Statistics of template		Statistics of model	
	residues	%	residues	%	residues	%	residues	%
Residues in most favored regions [A, B, L]	185	93.6	362	93.5	185	94.4	205	92.8
Residues in additional allowed regions [a, b, l, p]	23	5.6	25	6.5	11	5.6	16	7.2
Residues in generously allowed regions [~ a, ~ b, ~ l, ~ p]	0	0.0	0	0.0	0	0.0	0	0.0
Residues in disallowed regions	2	0.0	0	0.0	0	0.0	0	0.0
Number of non-glycine and non-proline residues	391	100	387	100	196	100	221	100
Number of end-residues (excl. Gly and Pro)	**4**		**2**		**1**		**2**	
Number of glycine residues (shown as triangles)	**33**		**24**		**26**		**17**	
Number of proline residues	**25**		**25**		**11**		**13**	
Total number of residues	**453**		**438**		**334**		**253**	

Significant values are in bold.

**Table 6:** PROCHECK, VERIFY3D and ERRAT evaluation statistics of *aflD* and *aflS*:

Protein		PROCHECK server				Verify 3D	ERRAT
		Most favoured regions	Additional allowed regions	Generally allowed regions	Disallowed regions	3D-1D score	Quality factor
*Afla*-S	Model	93.5%	6.5%	0.0%	0.0%	87.42%	96.40%
	Template	93.6%	5.6%	0.0%	0.0%	89.50%	96.55%
*Afla*-D	Model	92.8%	7.2%	0.0%	0.0%	93.68%	95.51%
	Template	94.4%	5.6%	0.0	0.0	98.72%	100%

#### Docking and interaction of compounds with 3D model of *aflD*, *aflS *and *aflR* of *A. flavus*

**Fig. 9:**
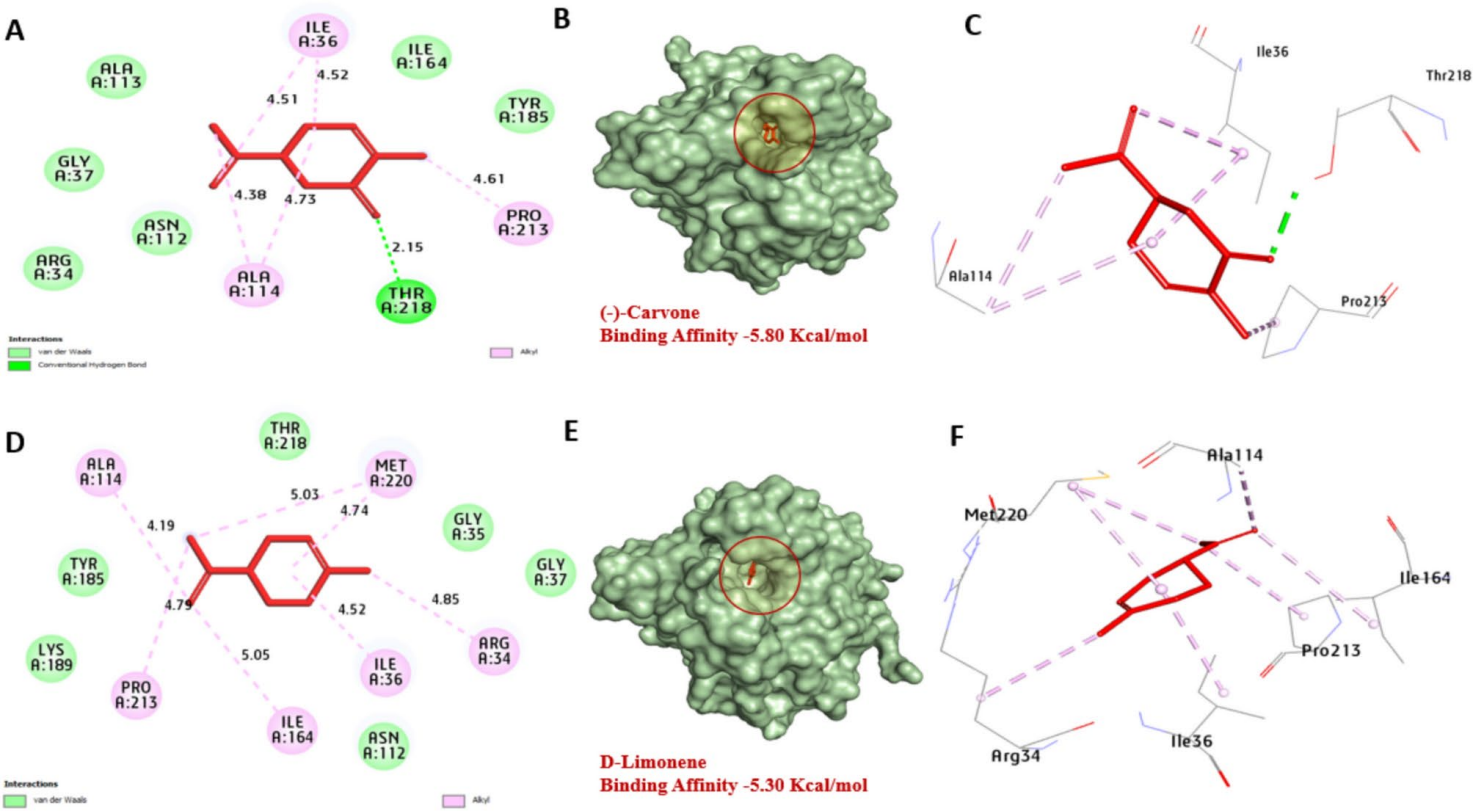
Molecular interactions of CEO’s active components with the amino acids in the 3D model of *aflD* are shown with the optimal binding mode. The ligand is depicted as red sticks, with [**A**, **B**, and **C**] representing (-)-Carvone and [**D**, **E**, and **F**] representing D-Limonene. The 2D interaction diagram highlights hydrogen bonds (green dashed lines) between the ligands and amino acids.

**Fig. 10:**
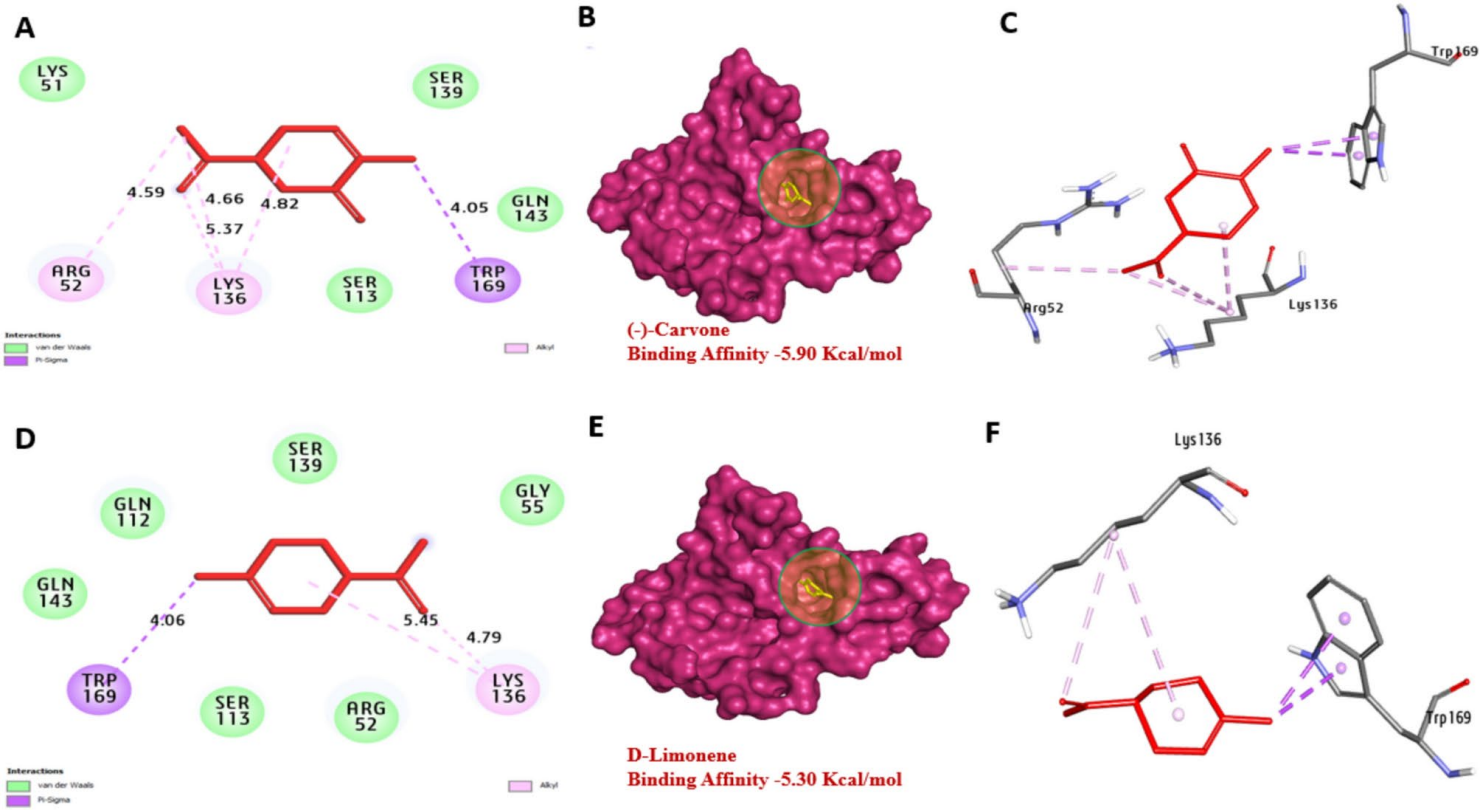
Molecular interactions of CEO’s active components with amino acids in the 3D model of *aflS* are shown with the optimal binding mode, where the ligand is represented as red sticks. Panels [**A**, **B**, and **C**] show (-)-Carvone, while [**D**, **E**, and **F**] depict D-Limonene. The 2D interaction diagram illustrates hydrogen bonds (green dashed lines) between the ligands and amino acids.

**Fig. 11:**
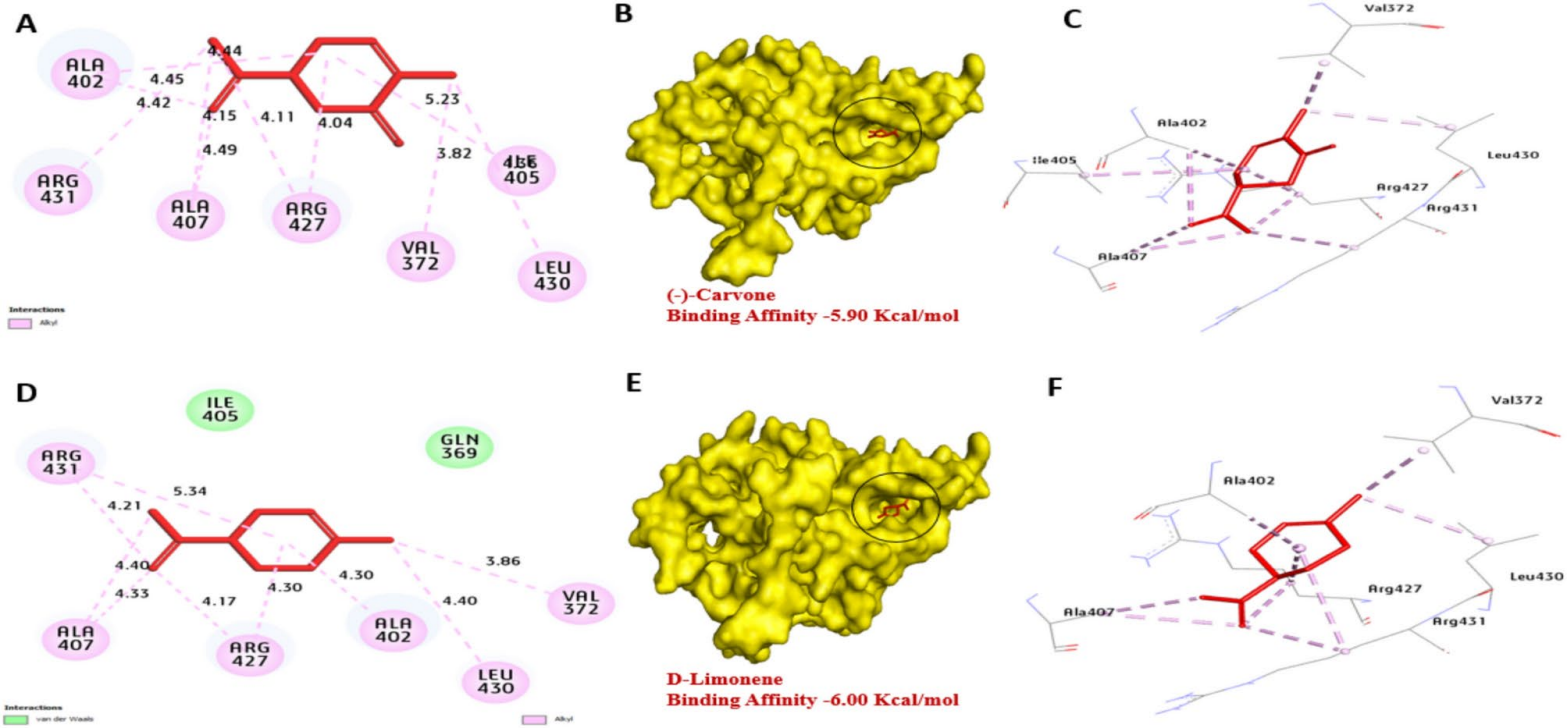
The molecular interactions of CEO’s active components with the amino acids in the 3D model of *aflD* are illustrated, showing the optimal binding mode. The ligand is depicted as red sticks, with [**A**, **B**, and **C**] corresponding to (-)-Carvone and [**D**, **E**, and **F**] to D-Limonene. The 2D interaction diagram highlights hydrogen bonds (represented by green dashed lines) between the ligands and amino acids.

The aflDgene is involved in the conversion of norsolorinic acid (NOR) to averantin, targeting this gene can disrupt the downstream steps in aflatoxin biosynthesis, ultimately leading to reduced aflatoxin production. Based on the docking analysis, *aflD* protein of *A. flavus* has a strong affinity for Carvone and D-Limonene with binding energies of − 5.80, and − 5.30 kcal/mol compared with (averantin − 5.10 kcal/mol) Table 7 (-)-Carvone formed one hydrogen bond with Thr218. Also, hydrophobic interactions including (Pi-alkyl) with Ala114, Ile36 and Pro213. Also, D-Limonene, not formed hydrogen bonds. In addition, hydrophobic interactions including (Pi-alkyl) with Met220, Arg34, Ile36, Ile164, Pro213, and Ala114. It can be observed that residues Thr218, Ala114, and Lys136 in the catalytic site enhance the binding affinity, Fig. 9. *aflS* protein binds to specific DNA sequences called aflatoxin response elements (AFREs) within the promoter regions in cluster genes, activating their transcription. Based on the docking analysis, *aflS* protein of *A. flavus* has a strong affinity for (-)-Carvone and D-Limonene with binding energies of − 5.80, and − 5.30 kcal/mol. No hydrogen bonds were performed with Carvone and D-Limonene. Also, hydrophobic interactions including (Pi-alkyl) with Lys136, Arg52 and Trp169 and (Pi-sigma) with Trp169 were performed with Carvone and D-Limonene. It can be observed that residues Lys136, Arg52, and Trp169 enhance the binding affinity by the formation of bonds. These promising compounds act as inhibitors and interfere with *aflR*'s proteins or disrupt its interaction involved in aflatoxin biosynthesis, Fig. 10. *aflR* protein is crucial for the expression of genes involved in various steps of aflatoxin biosynthesis. Based on the docking analysis, *aflR* protein of *A. flavus* has a strong affinity for Carvone and D-Limonene with binding energies of − 5.90, and − 6.00 kcal/mol. No hydrogen bonds were performed with Carvone and D-Limonene. Also, hydrophobic interactions including (Pi-alkyl) with Ile405, Leu430, Val372, Arg427, Ala407, Arg431 and Ala402. It can be observed that residues Ala402, Arg431, and Ile405 enhance the binding affinity by the formation of bonds, Fig. 11. Our findings align with those of Das et al.^45^, indicating a molecular interplay between eugenol and various amino acids of the Ver-1 gene product, chosen for its pivotal regulatory role in AFB1 biosynthesis. Consequently, Carvone and D-Limonene were investigated for their capacity to interact with the *aflD*, *aflS*, and *aflR* proteins of *A. flavus*, potentially through internal penetration and hydrogen bonding mechanisms that rely on the stereo-spatial configurations within the catalytic regions. This interaction could plausibly contribute to the inhibition of AFB_1_ biosynthesis.

**Table 7:** Molecular interactions of the target compounds with amino acids of 3D model of *aflD, aflS* and *aflR* of *A. flavus*:

No.	Protein	Ligand	3D structure	Hydrophilic interactions		Hydrophobic contacts		No. of H-Bonds	No. of total Bonds	Affinity kcal mol-1
				Residue (H-Bond)	Length	Residue (Bond type)	Length			
1	*aflD*	(-)-Carvonene		Thr218, (H- Bond)	2.15	Ala114, (Pi-alkyl) Ala114, (Pi-alkyl) Ile36, (Pi-alkyl) Ile36, (Pi-alkyl) Pro213, (Pi-alkyl)	4.73 4.38 4.52 4.51 4.61	1	6	− 8.80
2		D-Limonene		–	–	Met220, (Pi-alkyl) Met220, (Pi-alkyl) Arg34, (Pi-alkyl) Ile36, (Pi-alkyl) Ile164, (Pi-alkyl) Pro213, (Pi-alkyl) Ala114, (Pi-alkyl)	5.03 4.74 4.85 4.52 5.05 4.79 4.19	0	7	− 5.30
3	*aflS*	(-)-Carvonene		–	–	Lys136, (Pi-alkyl) Lys136, (Pi-alkyl) Lys136, (Pi-alkyl) Arg52, (Pi-alkyl) Trp169, (Pi-alkyl)	4.82 5.37 4.66 4.56 4.05	0	5	− 5.90
4		D-Limonene		–	–	Lys136, (Pi-alkyl) Lys136, (Pi-alkyl) Trp169, (Pi-sigma) Trp169, (Pi- alkyl)	4.76 5.45 4.06 2.05	0	4	− 5.30
5	*aflR*	(-)-Carvonene		–	–	Ile405, (Pi-alkyl) Leu430, (Pi-alkyl) Val372, (Pi-alkyl) Arg427, (Pi-alkyl) Ala407, (Pi-alkyl) Arg431, (Pi-alkyl) Ala402, (Pi-alkyl) Ala402, (Pi-alkyl) Ala407, (Pi-alkyl) Arg427, (Pi-alkyl)	5.23 4.36 3.82 4.04 4.04 4.42 4.42 4.45 4.11 4.04	0	10	− 5.90
6		D-Limonene		–	–	Leu430, (Pi-alkyl) Val372, (Pi-alkyl) Arg427, (Pi-alkyl) Ala407, (Pi-alkyl) Arg431, (Pi-alkyl) Arg431, (Pi-alkyl) Ala402, (Pi-alkyl) Ala407, (Pi-alkyl) Arg427, (Pi-alkyl)	4.40 3.86 4.30 4.17 4.30 4.40 4.33 4.17 5.34	0	9	− 6.00

## Conclusion

This study demonstrated the feasibility of coupling chitosan with caraway essential oil (CEO) to create eco-friendly antifungal bionanocomposites with enhanced activity compared to the individual effects of chitosan and CEO alone. The findings revealed that CEO-CSNPs significantly inhibit the growth of aflatoxigenic fungi and aflatoxin secretion more effectively than CEO alone and offering a promising natural alternative for food preservation. According to the molecular docking analyses, carvone and D-limonene, the major components of CEO, act as inhibitors and interfere with *aflR*'s proteins or disrupt their interaction involved in aflatoxin biosynthesis. These results indicate that CEO-CSNPs have considerable potential as a new antifungal preservative. They might help extend the shelf life of food by preventing contamination from fungi and aflatoxin.

## Data Availability

Any datasets used to support the findings of this study are available from the corresponding author upon reasonable request.
